# Role of echocardiography in clinical hypertension

**DOI:** 10.1186/s40885-015-0015-8

**Published:** 2015-06-17

**Authors:** Jae-Hwan Lee, Jae-Hyeong Park

**Affiliations:** Department of Cardiology in Internal Medicine, School of Medicine, Chungnam National University Hospital, Chungnam National University, Daejeon, Korea

**Keywords:** Hypertension, Cardiovascular risk, Echocardiography

## Abstract

Hypertension is a major and correctable cardiovascular risk factor. The correct diagnosis of hypertension and precise assessment of cardiovascular risk are essential to give proper treatment in patients with hypertension. Although echocardiography is the second-line study in the evaluation of hypertensive patients, it gives many clues suggesting bad prognosis associated with hypertension, including increased left ventricular (LV) mass, decreased LV systolic function, impaired LV diastolic function, and increased left atrial size and decreased function. Along with conventional echocardiographic methods, tissue Doppler imaging, three-dimensional echocardiography, and strain echocardiography are newer echocardiographic modalities in the evaluation of hypertensive patients in the current echocardiographic laboratories. Understanding conventional and newer echocardiographic parameters is important in the diagnosis and assessment of cardiovascular risk in hypertensive patients.

## Introduction

Cardiovascular diseases are major leading health problems in the world [1], and hypertension is a major risk factor for cardiovascular diseases and stroke which have significantly higher morbidity and mortality [2].

Although the echocardiographic examination is usually recommended as a second-line study in the evaluation of hypertensive patients, it is one of most commonly used imaging modality and has given insights into pathophysiology and clinical implications in patients with hypertension. It can detect anatomical and functional changes easily in a real-time, quick, and reproducible manner. Echocardiography is more sensitive for the detection of asymptomatic organ damage that can be used as a determinant of cardiovascular risk. So, it is important in the clinical management in selected hypertensive patients.

In this review, we want to describe the role of echocardiography in the evaluation of hypertensive patients.

## Review

### Recommendations of echocardiography in the current hypertension guidelines

In the 2013 ESH/ESC Guidelines for the management of arterial hypertension, echocardiography is the second-line study based on medical history, physical examination, and findings from routine laboratory tests [3]. The guidelines recommended performing echocardiographic examination in patients who are suspected with having left ventricular hypertrophy (LVH), left atrial (LA) dilatation, or concomitant heart diseases (class IIb: usefulness/efficacy is less well established by evidence/opinion, level C: consensus of opinion of the experts and/or small studies, retrospective studies, and registries).

The 2014 Canadian Hypertension Education Program (CHEP) guidelines recommended the echocardiographic use in the selected patients [4]. Routine echocardiographic study is not recommended in all patients with hypertension (grade D: recommendations are based on expert opinion alone). However, the echocardiographic examination for the evaluation of LVH is useful in selected patients to define the future cardiovascular risk (grade C: recommendations are based on trials that have lower levels of internal validity and/or precision, trials reporting invalidated surrogate outcomes, or results from non-randomized observational studies). Echocardiographic evaluation of LV mass and systolic and diastolic LV function is recommended in patients with hypertension suspected to have LV dysfunction or coronary artery disease (grade D). LV ejection fraction (LVEF) should be assessed objectively by an echocardiogram or nuclear imaging in patients with hypertension and evidence of heart failure (grade D).

The latest guideline of the Eighth Joint National Committee (JNC 8) did not mention about the use of echocardiography [5]. The previous guideline of JNC 7 discussed about the echocardiographic examination to detect LVH in the assessment of cardiovascular risk [6].

### Appropriateness of echocardiography in hypertension

Because the echocardiography is the most common and the first-line imaging study in many clinical scenarios, appropriateness is an important issue in reducing the cost of inappropriate use of echocardiography. The American College of Cardiology Foundation published the appropriate use criteria for echocardiography in cooperation with the American Society of Echocardiography and along with key specialty and subspecialty societies [7]. They have analyzed many clinical scenarios and categorized three groups according to the appropriate use score: appropriate (median 7–9), uncertain (median 4–6), and inappropriate (median 1–3).

The use of echocardiography as an initial evaluation of suspected hypertensive heart disease is appropriate (appropriate use score 8). Also, echocardiography is appropriate in patients with prior test result that is concerning for heart disease or structural abnormality (appropriate use score 9). Initial evaluation of known or suspected heart failure (HF), systolic or diastolic, based on symptoms, signs, or abnormal test results, is appropriate (appropriate use score 9). Re-evaluation of known HF with a change in clinical status or cardiac examination without a clear precipitating change in medication or diet is appropriate (appropriate use score 8).

**Figure 1:**
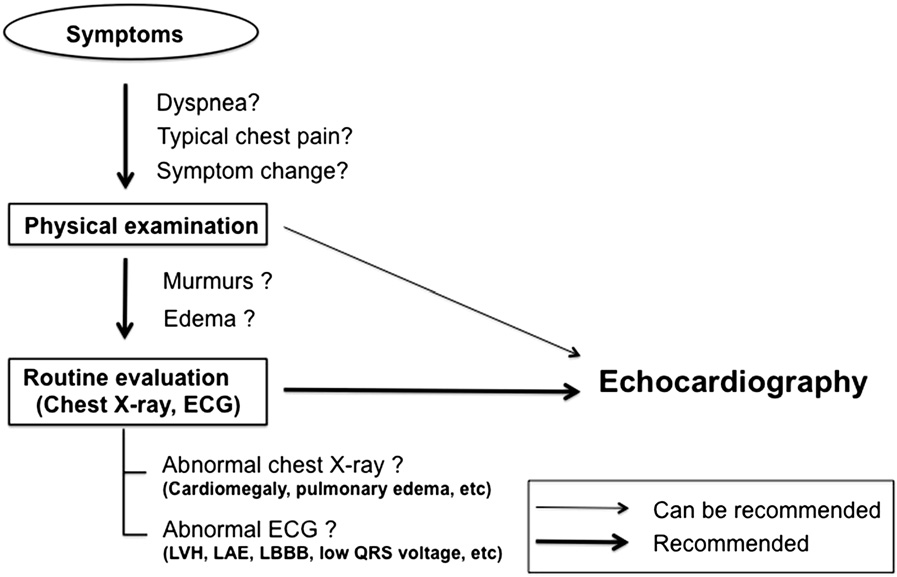
**Diagnostic flowchart in the evaluation of hypertension.** Usually, the echocardiography is a second-line test. Bold solid lines refer to routine recommendation. Solid lines refer to tests that ‘can be recommended’.

However, routine evaluation of systemic hypertension without symptoms or signs of hypertensive heart disease is inappropriate (appropriate use score 3). Initial echocardiographic evaluation of ventricular function with no symptoms or signs of cardiovascular disease is inappropriate (appropriate use score 2). It is also inappropriate for the evaluation of LV function with prior ventricular function evaluation showing normal function in patients in whom there has been no change in clinical status or cardiac examination (appropriate use score 1). However, re-evaluation of known hypertensive heart disease without a change in clinical status or cardiac examination is uncertain (appropriate use score 4). The usual recommendations of echocardiographic examination in the evaluation of arterial hypertension are summarized in Table 1, and simplified algorithm of the echocardiography is summarized in Figure 1.

**Table 1:** **Clinical situations when the echocardiography is recommended in the evaluation and treatment of arterial hypertension**

**Clinical situations**	**Signs**
Heart failure is suspected	-Symptoms: exertional dyspnea, orthopnea, generalized edema, etc.
	-Abnormal physical examination: cardiac murmurs, pretibial pitting edema, etc.
	-Abnormal ECG results: left ventricular hypertrophy, left atrial enlargement, left bundle branch block, pathologic Q waves, poor R progression, atrial fibrillation etc.
	-Abnormal chest X-ray findings: cardiomegaly, pulmonary edema, pleural effusion, etc.
Structural heart disease is suspected	-Symptoms: exertional dyspnea, orthopnea, etc.
	-Abnormal physical examination: cardiac murmurs, pretibial pitting edema, etc.
	-Abnormal ECG results: left ventricular hypertrophy, right ventricular hypertrophy, left atrial enlargement, right atrial enlargement, etc.
	-Abnormal chest X-ray findings: cardiomegaly, pulmonary edema, pleural effusion, etc.
Ischemic heart disease is suspected	-Symptoms: typical chest pain, exertional dyspnea, etc.
	-Abnormal ECG results: significant ST changes, pathologic Q wave, etc.
Refining cardiovascular risk

### Echocardiographic evaluations

#### Left ventricular mass and geometry

The evaluation of LVH with measuring mass and geometry is the major role of echocardiography in hypertensive patients. In the detection of LVH, echocardiographic assessment is more sensitive than electrocardiography [8,9]. Echocardiography is a good diagnostic tool in the determination of overall cardiovascular risk and helps in the selection of appropriate antihypertensive therapy [9].

The calculation of LV mass is based on subtraction of the LV cavity volume from the volume enclosed by the LV epicardial surface. To measure proper LV mass, precise evaluation of thicknesses of the interventricular septum and LV posterior wall in addition to the dimension of the interventricular cavity is needed. The LV mass has been measured using this equation that has been recommended by the American Society of Echocardiography (ASE) which is derived from two-dimensional linear LV measurements [10];

$$ \mathrm{Left}\;\mathrm{ventricular}\;\mathrm{mass}\;\left(\mathrm{gram}\right)=0.8\times 1.04\times \left[{\left(\mathrm{LVIDd}+\mathrm{PWTd}+\mathrm{SWTd}\right)}^3-{\mathrm{LVIDd}}^3\right]+0.6 $$

where LVIDd is the LV internal dimension at end diastole, PWTd is the LV posterior wall thickness at end diastole, and SWTd is interventricular septal wall thickness at end diastole. Although this ASE-recommended formula showed excellent correlation with necropsy study (*r* = 0.90, *p* < 0.001) [11], even small errors in the measurement are magnified and lead to big differences [10].

**Figure 2:**
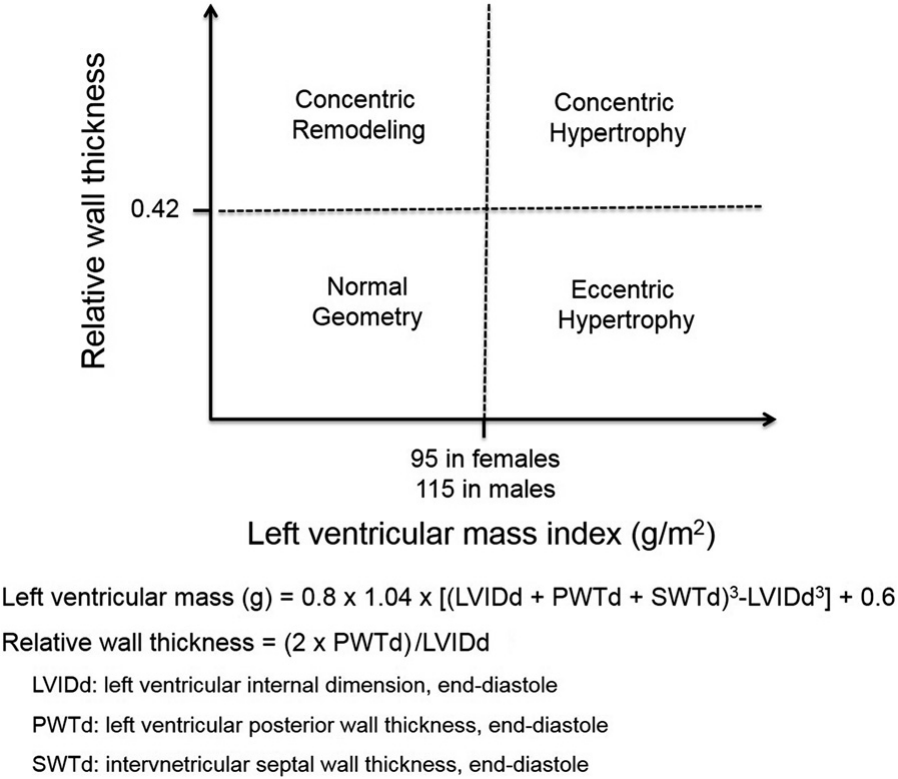
**Classification of hypertensive patients according to the presence of left ventricular hypertrophy and the relative wall thickness.**

The relative wall thickness (RWT) is calculated by the formula (2 × PWTd)/LVIDd [10]. RWT can categorize the LVH as either concentric (RWT greater than 0.42) or eccentric (RWT less than 0.42). With certain cutoff values for LV mass have been widely accepted for the presence of LVH (125 g/m^2^ for men and 110 g/m^2^ for women), patients with hypertension can be classified into four subgroups (Figure 2) [10,12]. LV geometric pattern provides additional prognostic information in hypertensive patients. Concentric hypertrophy is associated with increased cardiovascular events after adjustment for other cardiovascular risk factors including LV mass [13]. Also, concentric hypertrophy showed the greatest mortality risk in patients suspected with coronary artery disease [14].

**Figure 3:**
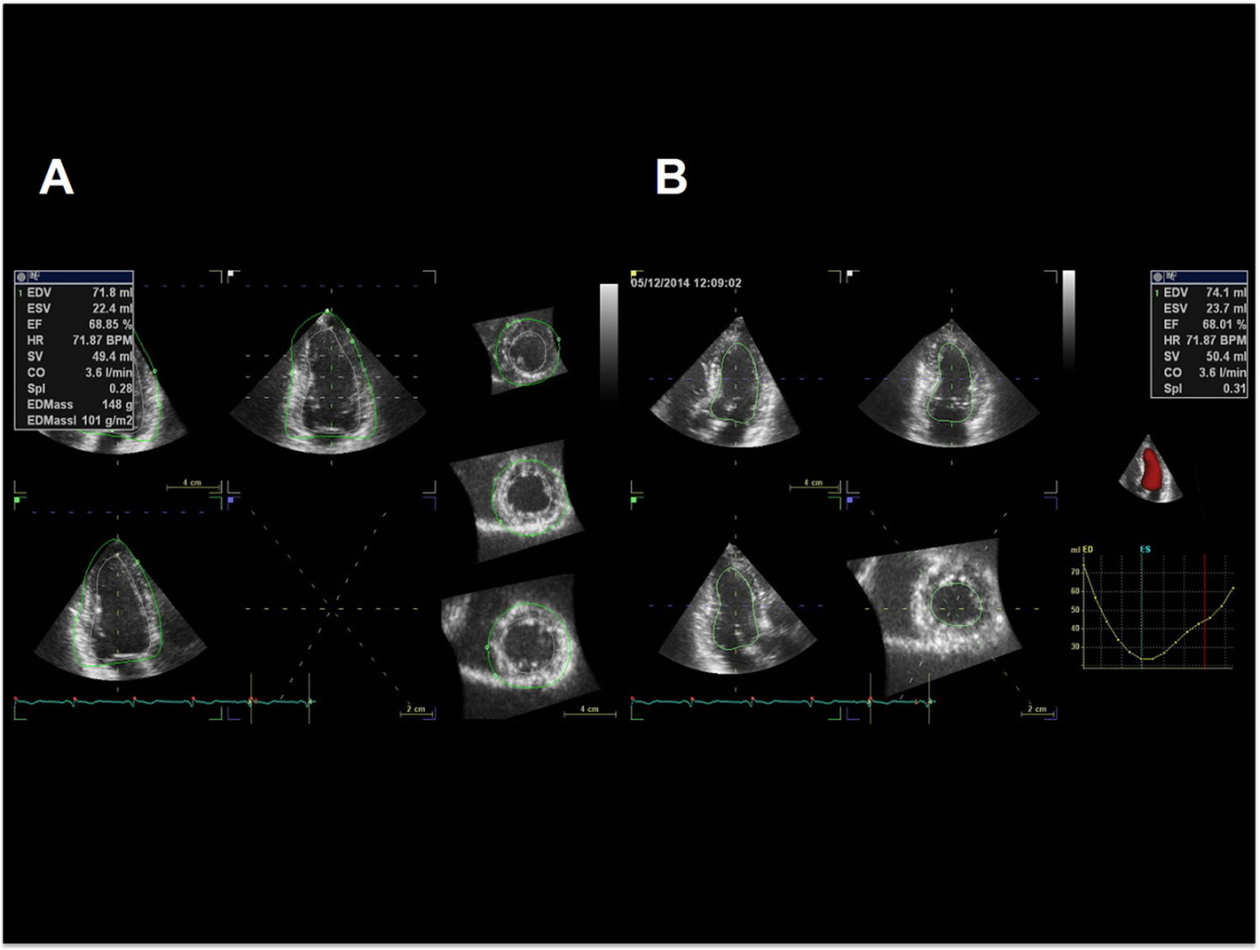
**Examples of calculation of left ventricular mass (A) and volumes (B) by three-dimensional echocardiography.**

Three-dimensional echocardiography has been introduced to assess LV mass (Figure 3A) [15,16]. Because M-mode and two-dimensional echocardiographic techniques have many limitations, three-dimensional echocardiography provides more precise measurements theoretically. Real-time three-dimensional echocardiographic measurement of LV mass showed an excellent correlation (*r* = 0.95, *p* < 0.001) with measurement by magnetic resonance imaging [16]. Real-time three-dimensional measurement showed better degree of agreement than M-mode or two-dimensional calculation of LV mass. Also, inter- and intraobserver variability were lower in the real-time three-dimensional echocardiographic method. Because of these advantages over conventional two-dimensional echocardiography, three-dimensional echocardiographic measurement can be a technique of choice in the studies of the regression of LVH with antihypertensive medications.

### Left ventricular systolic function

Rapid and reliable assessment of LV systolic function is another advantage of the echocardiography. Although coronary artery disease is the most common cause of LV systolic dysfunction, arterial hypertension is a possible cause of functional impairment of the LV. Echocardiography can give information about the possibility of coronary artery disease in addition to calculation of LV function.

**Figure 4:**
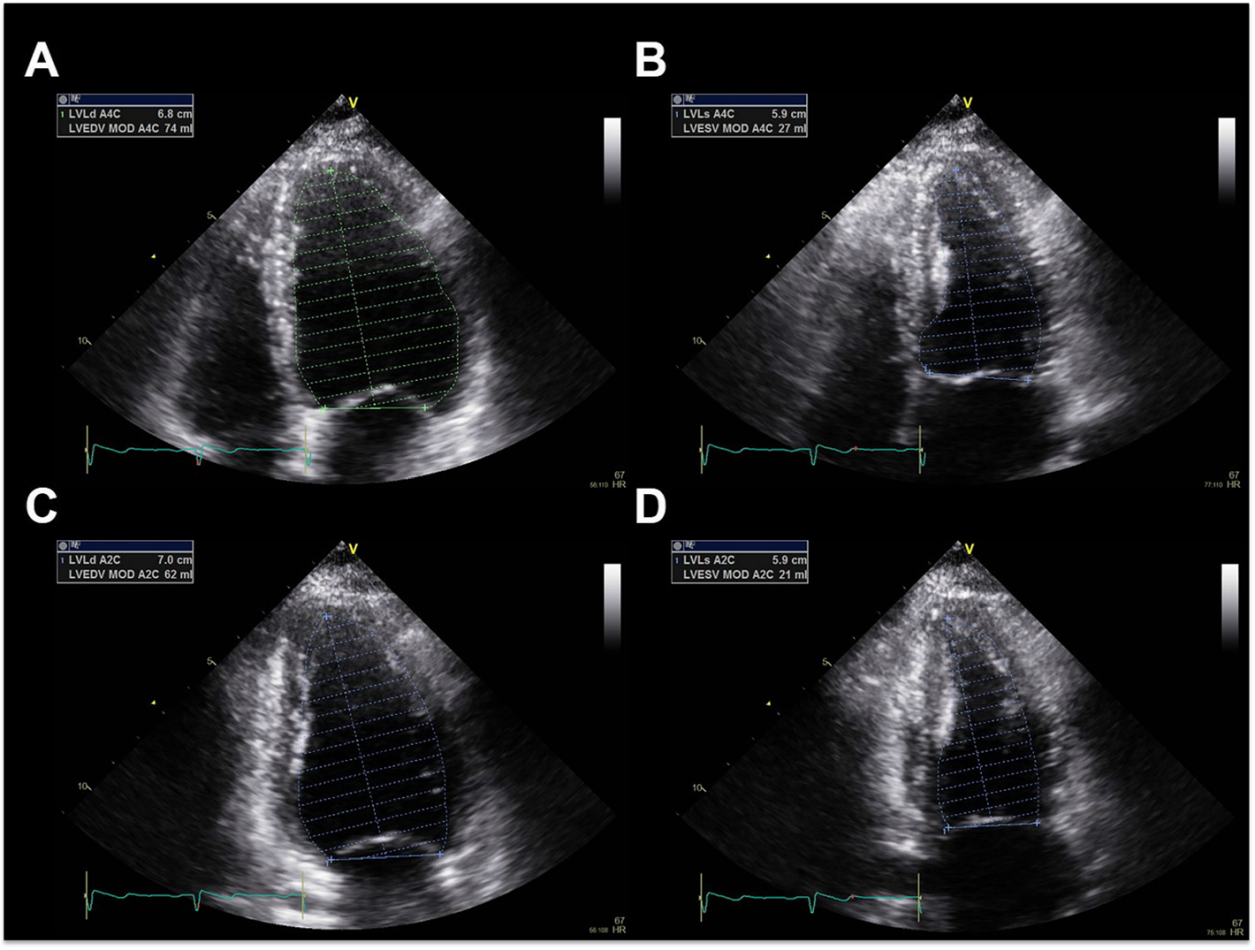
**Two-dimensional calculations for volume calculations using biplane methods of disks (modified Simpson’s method) in apical 4 chamber (A and B) and apical 2 chamber views (C and D).** Ejection fraction can be measured from the division of the stroke volume (subtraction of end-systolic volume from end-diastolic volume) by end-diastolic volume.

Several echocardiographic indices have been introduced to estimate LV systolic function. Among them, LVEF is the most used and the most practical systolic index that has been used as a prognostic factor in various cardiovascular diseases [16,17]. It can be calculated from the ratio between the volume ejected during systole over the end-diastolic volume of the LV. Echocardiographic measurement of ejection fraction is usually done using modified Simpson’s method (Figure 4) [10,18]. A normal LVEF is more than 55% in adults [10].

Three-dimensional echocardiographic measurements of LV volumes have been available with technical improvement (Figure 3B) [19-21]. Three-dimensional measurement does not require the assumption that the LV is a prolate ellipse, and it can measure LV volumes as it is. So, three-dimensional echocardiographic measurement has advantages over the calculation of LV volumes in patients with regional wall motion abnormalities or LV aneurysms. Three-dimensional echocardiographic measurements have been shown high degree of agreement with the measurements by cardiac magnetic resonance imaging [22-24]. Moreover, these studies demonstrated significant increases in reproducibility over conventional two-dimensional echocardiographic measurements.

Because impairment of LV long-axis function occurs at the early stages in many cardiac diseases, its assessment provides a very useful index in the evaluation of hypertensive patients [25]. The LV longitudinal function can be assessed with atrioventricular plane displacement that was abnormal in hypertensive patients without overt systolic dysfunction [26].

Tissue Doppler imaging is the new echocardiographic modality to measure mitral annular movement. Mitral annular velocity was decreased in hypertensive patients with normal ejection fraction [27], and it can be used to detect subclinical LV systolic dysfunction [28]. Measurements of myocardial function by strain echocardiography are newer indices that have advantages over other conventional echocardiographic measurements like LVEF. Strain can measure global and regional myocardial function, objectively. Two-dimensional speckle-tracking echocardiography analyzes myocardial deformation by tracking of natural acoustic markers that are generated from the interactions between ultrasound and myocardium. These natural acoustic markers are referred to as speckles [29,30]. Although there are several echocardiographic algorithms tracking the speckles, they are able to get angle-independent and multi-directional (longitudinal, radial, and circumferential) strain values [31]. Moreover, inter- and intraobserver variability of two-dimensional strain echocardiography are better than tissue Doppler values [32].

Myocardial strain can detect subclinical organ damage earlier than other conventional echocardiographic parameters. Kang et al. [33] reported longitudinal strain was decreased in hypertensive patients with normal LV systolic function, and it was correlated with serum level of tissue inhibitor of matrix metalloproteinase-1 level, a marker of myocardial fibrosis.

**Figure 5:**
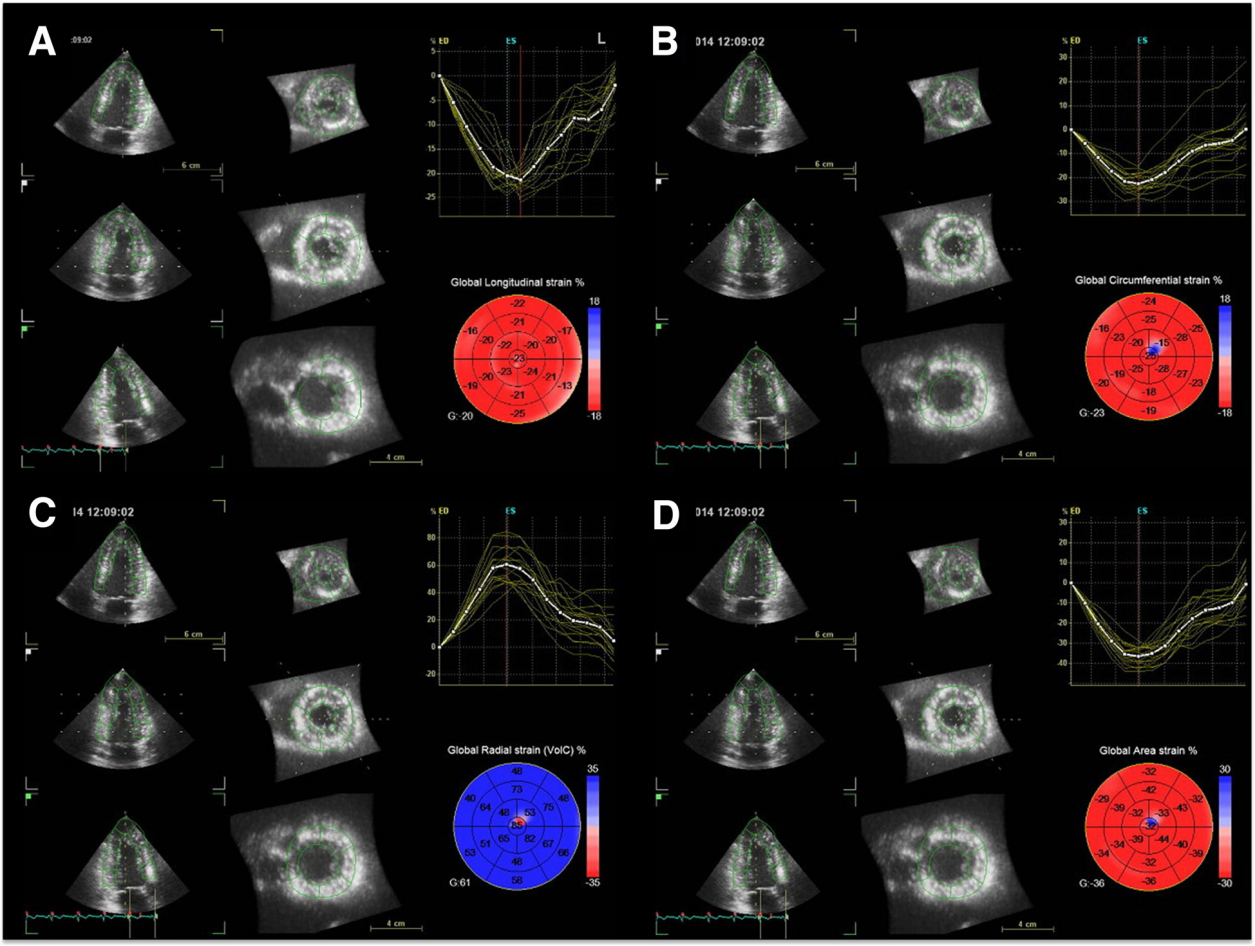
**Multiplanar reconstruction images are obtained automatically from single volumetric echocardiographic data.** Global longitudinal **(A)**, circumferential **(B)**, radial **(C),** and area strains **(D)** can be measured as well as regional segmental values at one analysis.

Three-dimensional strain echocardiography has been developed by technological advancement, and it can track the motion of myocardial speckles stereoscopically [34]. Analysis of the whole LV from a single volume data is the main advantage of three-dimensional strain echocardiography (Figure 5). It can measure longitudinal, circumferential, radial, and area strain and can also reduce the time duration required for analysis [35]. Circumferential strain by three-dimensional echocardiography showed better correlation with LVEF than with global longitudinal strain and radial strain, especially in patients with preserved LV function [36]. Recent studies reported that global area strain is the most reliable surrogate of LVEF [37,38]. Global area strain by three-dimensional strain echocardiography was precociously decreased in hypertensive patients with normal LV systolic function [39]. Three-dimensional echocardiographic strain analysis also can measure regional myocardial function.

### Left ventricular diastolic function

Diastole is a period to fill the LV, and normal diastolic function is required to fill the cardiac chambers without abnormally elevated pressure [40]. The diastole includes four sub-phases: isovolumetric relaxation, early rapid ventricular filling, diastasis, and atrial contraction. Diastole is not a passive process, and it starts after LV contraction [41]. The ventricular myocardium is compressed and twisted during LV systole. The compressed and twisted myocardium was relaxed and unloads the energy required during diastole. Intra-ventricular pressure drops rapidly during the first isovolumetric relaxation period. This relaxation makes a pressure difference between the LA and ventricle that forced the rapid blood flow through the mitral valve and fills the LV rapidly. During diastasis, blood flow continues through the mitral valve. The atrial contraction is the last phase of the diastole, and blood is pumped by the muscular contraction of the atrium.

Although LV diastolic dysfunction is associated with increased mortality in middle-aged and elderly adults [42], the estimation of LV diastolic function is more difficult than the measurement of systolic function [43]. A comprehensive assessment of LV diastolic function should estimate LV filling pressure.

Echocardiography is a useful imaging tool to measure LV diastolic function. Several echocardiographic modalities can be used to estimate LV filling pressure. Increased LA size and volume is one of indicator of increased LV filling pressure [44,45]. Enlarged LA diameter was found more than 20% of hypertensive patients in a large-scale study including a total of 2,500 uncomplicated essential hypertensives [46]. Enlarged LA can present long-standing elevated LV filling pressure, and increased LA size and volume were associated with poor long-term mortality and morbidity [47,48].

**Figure 6:**
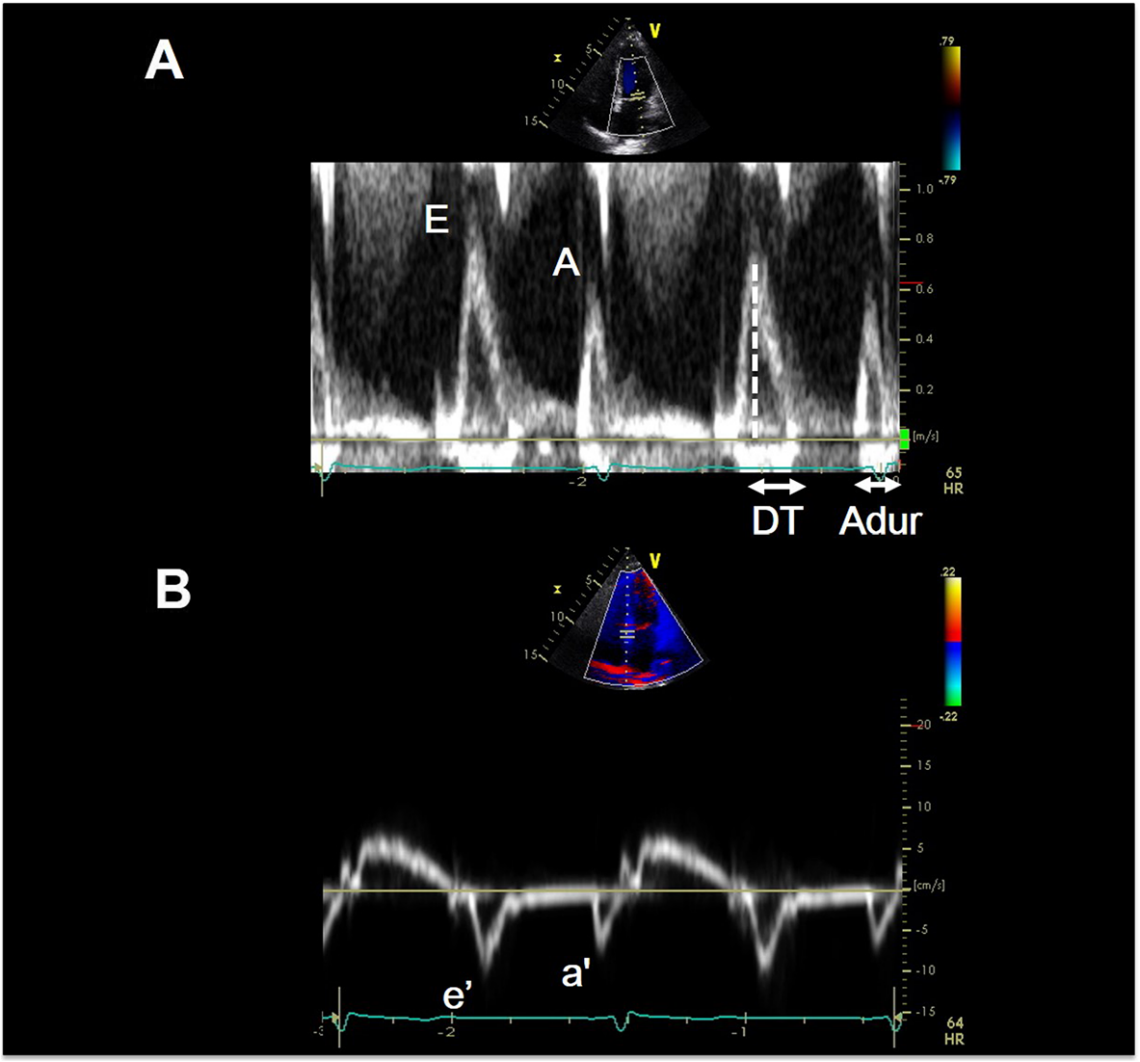
**Left ventricular diastolic function can be assessed with mitral inflow velocity and mitral annular velocity.** Mitral inflow velocities obtained by pulsed-wave Doppler technique **(A)** and their schematic diagram. Peak mitral inflow velocity during early diastole (E wave), peak mitral inflow velocity at atrial contraction (A wave), mitral deceleration time (DT), duration of A wave (Adur). Mitral annular velocities obtained by tissue Doppler echocardiography with e’ velocity as the early diastolic velocity and a’ velocity as the late diastolic velocity **(B)**.

Mitral inflow pattern by pulsed-wave Doppler technique is another maker of diastolic function (Figure 6A). Isovolumic relaxation time, ratio of E and A velocities, deceleration time of E velocity, and duration of A wave can be used to assess diastolic dysfunction [49]. However, these velocities can be influenced by multiple factors including age, heart rate and rhythm, cardiac output, mitral annular size, and LA function [49].

Mitral annular velocity can be assessed by pulsed-wave Doppler of mitral annulus from tissue Doppler imaging (Figure 6B). Because e’ velocity measured by tissue Doppler imaging is inversely proportionate to the relaxation time constant (tau) only, whereas transmitral E velocity is proportionate to the ratio between LA pressure and tau, the ratio E/e’ can be a good indicator of LA pressure [50-52], and it is generally the most feasible marker for estimation of LA filling pressure. Several validation studies have showed good correlation of this ratio with LA filling pressure, and the prediction of normal filling pressure is most reliable when the ratio is <8. When the ratio is >15, it is an indicator of increased LA filling pressure [49,52].

However, the E/e’ ratio has pitfalls in the assessment of LA filling pressure [53]. The E/e’ ratio had a poor correlation with LA pressures in patients with advanced systolic heart failure [54]. Situations where the use of E/e’ may be unreliable in tachycardia with fusion of mitral E and A velocities, significant valvular heart disease (significant mitral regurgitation, significant mitral stenosis, and aortic regurgitation), and presence of left bundle branch block [53].

Diastolic stress echocardiography using exercise stress can detect hemodynamic consequences of exercise-induced increase in diastolic filling pressure noninvasively [55]. It can detect subclinical diastolic dysfunction and can be valuable in patients with unexplained dyspnea [55].

### Left atrial size and function

LA enlargement is commonly associated with systemic arterial hypertension in patients without significant valvular heart disease, and it is associated with overweight, LVH, and metabolic syndrome [46]. LA size can be calculated with parasternal long-axis view at end systole along its greatest dimension, trying to avoid foreshortening it. Normal LA diameter is 2.7 ~ 3.8 cm for female and 3.0 ~ 4.0 cm for male [10].

**Figure 7:**
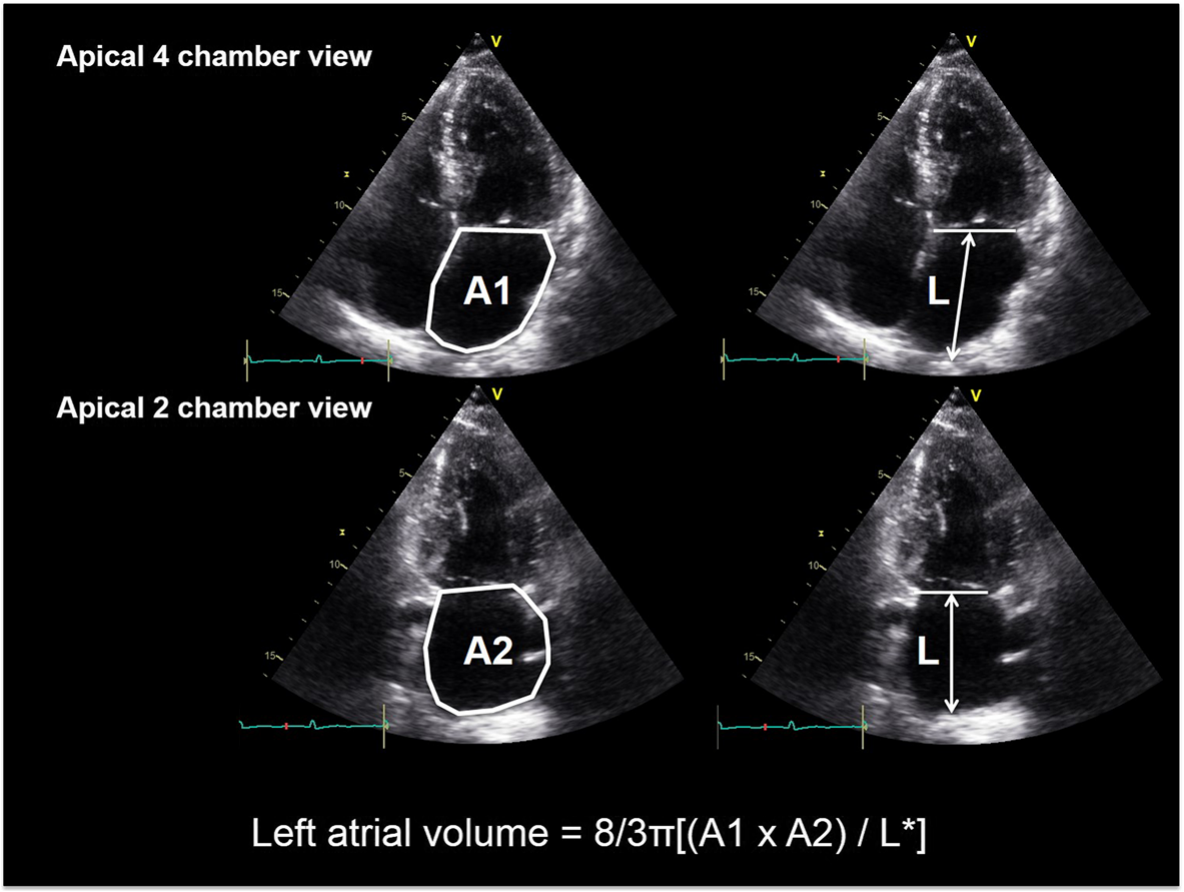
**Measurement of left atrial volume from area-length method using apical 4 chamber (A1) and apical 2 chamber (A2) views at ventricular end systole.** L* is the shortest of either the apical 4 chamber or apical 2 chamber length.

LA volume can be assessed by two-dimensional echocardiography (Figure 7). Normal LA volume is lower than 28 ml/m^2^ [10], and enlarged LA is one of the poor prognostic markers. Increased LA size and volume can reflect the diastolic dysfunction in hypertensive patients and can be used as one of indicators of cardiovascular morbidity and mortality [45]. Volume more than 34 ml/m^2^ is associated with poor prognosis including death, heart failure, atrial fibrillation, and ischemic stroke [56,57]. Because the LA does not show rapid reverse remodeling with treatment, its size or volume is an inadequate marker of therapeutic response.

LA function is important in the regulation of LV filling by reservoir, conduit, and booster pump functions [58]. LA reservoir and conduit functions are essential during the early diastolic phase, and booster pump function is needed in active filling of the LV during the late diastolic phase. The echocardiographic assessment of LA function is relatively difficult due to its anatomical shape [59]. Tissue Doppler imaging has been introduced to measure LA booster function. The positive wave during systole (SRs) reflects the reservoir function, the negative wave during early diastole (SRe) indicates the conduit function, and the negative wave during late diastole (SRa) represents the booster pump function [60,61]. LA strain and strain rate can detect subclinical atrial dysfunction in patients with hypertension [61]. However, strain and strain rate assessed by tissue Doppler imaging have limitation about angle dependency, and the deformation characteristics of the LA in hypertensive patients are still controversial [62].

Two-dimensional speckle-tracking echocardiography has been used in the assessment of LA function in a noninvasive, simple, and reproducible manner [63]. Hypertensive patients with decreased LA function assessed by speckle-tracking echocardiography have poor prognosis [64,65]. Also, strain and strain rate from two-dimensional speckle-tracking echocardiography may reflect decreased LA conduit function and increased booster pump function in hypertensive patients with LVH [63].

Analysis of LA appendage can give indirect information about LA function, and impaired LA appendageal function in patients with non-dipper compared to dipper hypertensive patients [66]. Although the strain measurement of LA is not recommended in routine clinical practices, it can be a good indicator of LA function.

Three-dimensional echocardiography has been introduced in the measurement of LA size and volume, and it gives many advantages over two-dimensional echocardiography [67]. Real-time three-dimensional echocardiography can provide a reproducible assessment and passive LA function by volumetric cyclic changes [68]. Its measurement of LA volume showed well correlation with LA volume by multi-detector computerized tomography [69]. It may be superior to the two-dimensional echocardiography because of its higher sensitivity to volume changes [68].

### Other echocardiographic findings in hypertensive patients

Secondary pulmonary hypertension can be resulted from increased LA pressure transmitted to pulmonary circulation. Also, HF with preserved ejection fraction is a common cause of pulmonary hypertension [70]. Echocardiography can estimate pulmonary arterial pressure with tricuspid regurgitation velocity or right ventricular outflow track flow profile [71]. Pulmonary arterial systolic pressure can be assessed by adding pressure gradient through right heart chambers (calculated with the Bernoulli equation) to right atrial pressure [72].

$$ \mathrm{PASP}=\mathrm{RVSP}=\mathrm{RAP}+4\times \mathrm{T}\mathrm{R}\;{\mathrm{Vmax}}^2 $$

Where PASP is the pulmonary artery systolic pressure, RVSP is the right ventricular systolic pressure, TR Vmax is the maximal tricuspid regurgitant velocity, and RAP is the right atrial pressure.

Right ventricular outflow tract acceleration time is the time from the beginning of the right ventricular ejection until the maximum of the systolic velocity [73]. This acceleration time is about 140 ms in normal people, and it shortens in patients with pulmonary hypertension (about 80 ms).

Because systemic arterial hypertension is also a risk factor of atherosclerosis, atherosclerotic heart diseases can be seen during echocardiographic examination. Ischemic heart disease, dilatation of ascending aorta, and aortic valve sclerosis or stenosis can be found. Presence of regional wall motion abnormalities or ventricular aneurysm is associated with ischemic coronary artery diseases. Stress echocardiography with using exercise protocol or dobutamine stress can detect ischemic heart diseases more sensitively. Dilatation of ascending aorta is associated with increased arterial stiffness and LV mass, and its reported incidence was 17% in hypertensive patients [74]. Calcific aortic valve diseases are usual manifestations of atherosclerosis. The presence of aortic valve sclerosis is associated with poor clinical outcomes [75,76].

## Conclusion

Because echocardiography can detect cardiac morphologic and hemodynamic change caused by systemic arterial hypertension, echocardiography is a powerful tool for the evaluation of target organ damage, which is essential for the evaluation of cardiovascular risk. Although echocardiography is not an essential first-line imaging study, echocardiography is an excellent tool for the assessment of future cardiovascular risks. Because of its non-invasiveness and easy accessibility, it is also a widely and most commonly used imaging modality in the cardiology practice. However, conventional echocardiography has many pitfalls in the interpretation of several echocardiographic parameters. To overcome this limitation, physicians should be aware of the pitfalls of conventional echocardiographic parameters. Second, doctors should analyze and interpret echocardiographic findings in conjunction with other findings from physical examination and routine examinations. Third, it is worthwhile for medical practitioners to learn other newer echocardiographc modalities. Aside from conventional echocardiographic modalities, newer echocardiographic methods including tissue Doppler imaging, strain echocardiography, or three-dimensional echocardiography also have been introduced to evaluate hypertensive patients providing valuable information about the extent of cardiac damages thus helping us to give better treatment.

## Abbreviations

LV: left ventricle
LA: left atrium
LVID: left ventricular internal dimension
IVS: interventricular septum
LVPW: left ventricular posterior wall
LVH: left ventricular hypertrophy
LVEF: left ventricular ejection fraction
